# Supplementary material for: The causal role of circulating inflammatory markers in osteoporosis: a bidirectional Mendelian randomized study
Source: Front Immunol. 2024 Jul 18;15:1412298. doi: 10.3389/fimmu.2024.1412298 (PMC11291241; doi:10.3389/fimmu.2024.1412298)
Supplement: Supplementary file 1 [file DataSheet_1.zip › supplementary material/CIM-OSTEOPOROSIS /GCST90274786.csv_forest.pdf]

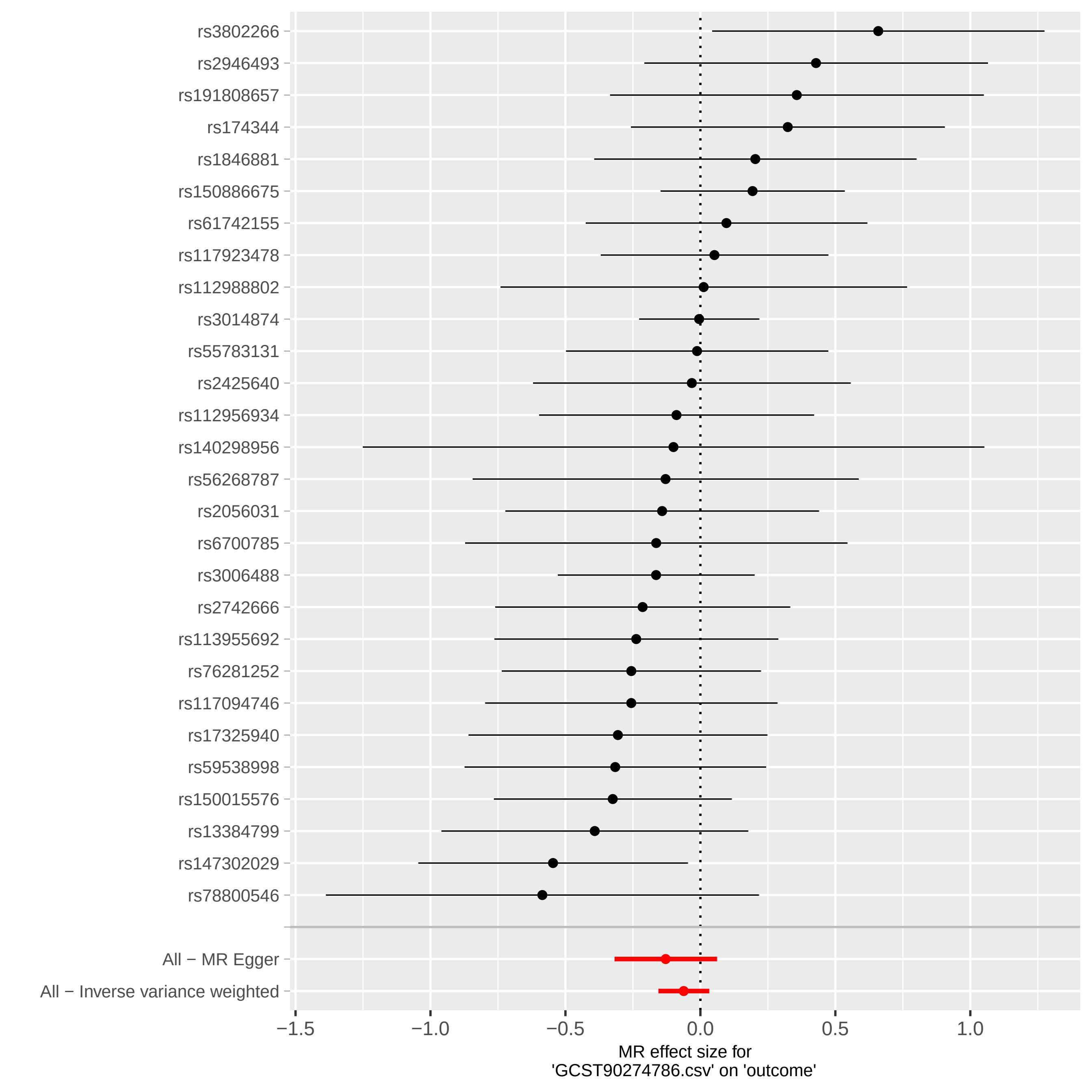
rs3802266
rs2946493
rs191808657
rs174344
rs1846881
rs150886675
rs61742155
rs117923478
rs112988802
rs3014874
rs55783131
rs2425640
rs112956934
rs140298956
rs56268787
rs2056031
rs6700785
rs3006488
rs2742666
rs113955692
rs76281252
rs117094746
rs17325940
rs59538998
rs150015576
rs13384799
rs147302029
rs78800546
All − MR Egger
All − Inverse variance weighted
−1.5
−1.0
−0.5
0.0
0.5
1.0
MR effect size for
'GCST90274786.csv' on 'outcome'
